# Deciding the Cause of Death in a Victim of a Road Traffic Accident Became Difficult Due to Interventions: A Case Report

**DOI:** 10.7759/cureus.33716

**Published:** 2023-01-12

**Authors:** Ashok Kumar Rastogi, Tarun Kumar, Amit Patil, Binay Kumar

**Affiliations:** 1 Forensic Medicine and Toxicology, All India Institute of Medical Sciences, Patna, IND; 2 Pathology/Lab Medicine, All India Institute of Medical Sciences, Patna, IND

**Keywords:** subendocardial hemorrhage, arrhythmia, septal contusion, conduction system, atrioventricular node

## Abstract

Though uncommon, puncture injury to the heart can occur during cardiac resuscitation and when inserting a lifesaving drug directly into the left ventricle of the heart. Utmost precaution must be taken to avoid damaging the conduction system of the heart, particularly the nodal part, as it can cause cardiogenic shock, arrhythmia, and sudden death. Our index case report describes a 55-year-old male who was fatally injured after being hit by a truck while riding his bike. The autopsy revealed multiple puncture injuries to the atrioventricular node area of the septum, observed on both sides of the interatrial septal wall surface. Histopathological examination also showed subendocardial hemorrhage around the atrioventricular nodal area. The nature of the injuries made identification of the cause of death difficult.

## Introduction

Cardiac injury can be caused by various inflictions, such as mechanical trauma (e.g. road accidents), cardiac interventions, and pathological conditions [1]. Injury to the conductive system of the heart may cause sudden cardiac arrest, arrhythmias, or even sudden death [2-5]. In patients with severe trauma or multiple fatal injuries, it can be difficult to identify the exact cause of death, especially if medical interventions such as resuscitations are applied and produced unwanted injuries or artifacts on the body. Punctures in the cardiac cavities usually do not cause death, as during resuscitation, lifesaving drugs are directly inserted into the heart chamber via a needle puncture. Injuries to the atrioventricular (AV) node, induced conductive system failure, and arrhythmia are considered to be the cause of death [5]. Commotio cordis (agitation of the heart) is a phenomenon in which blunt force impact causes sudden death without any structural damage to cardiac tissue. Commotio carditis is caused by abnormal cardiac rhythm resulting from lethal disruption. The AV node is situated at the lower back section of the interatrial septum near the opening of the coronary sinus and it sends the electrical impulse from the atria to the ventricles [6].

## Case presentation

**Figure 1:**
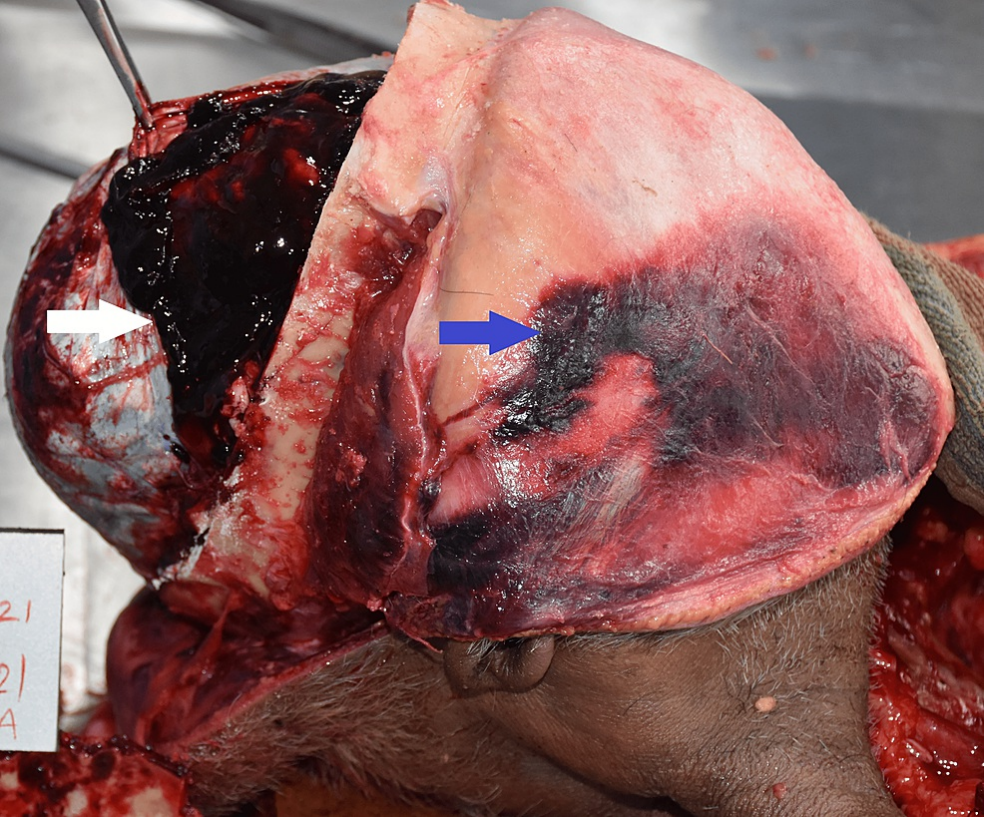
Scalp hematoma (blue arrow) and subdural hematoma seen after tearing the dura over the right side of the brain (white arrow)

**Figure 2:**
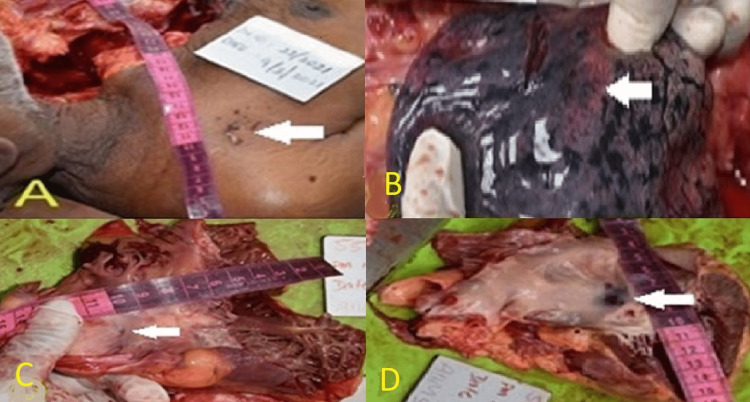
Autopsy findings (A) Multiple puncture marks on the right-side chest just below the lateral end of the clavicle. (B) Multiple puncture marks over the middle lobe of the right lung surface. (C) Interatrial septal puncture mark at the level of the atrioventricular node on the right atrial side. (D) Interatrial septal puncture mark with a contusion on the left atrial side.

The history of the incident was given by the relatives of the deceased. A 55-year-old male met with an accident and sustained severe craniocerebral and other injuries (Tables 1, 2 and Figure 1). He was admitted to the hospital. The accident was between a bike and a heavy vehicle lorry, and the deceased was a bike rider. The treating surgeon declared him dead after six hours of admission to the hospital. Head injury was noted in the hospital records and no surgical intervention was given due to the short span of survival. At the terminal stage, the treating surgeon tried to revive the patient by cardiopulmonary resuscitation. Then the body of the deceased was sent to the mortuary for an autopsy examination (Figure 2).

**Table 1:** Craniocerebral injuries

S. No.	Details of the injuries
1	Scalp hematoma of size 17 cm x 8 cm over the right lateral side of the head involving the right parietal, temporal, and occipital region, situated 4 cm above the right mastoid prominence.
2	Contusion of size 6 cm x 5 cm over right temporalis muscle situated 2 cm above the tip of the right mastoid process.
3	Fissured fracture of length 12 cm starting at the right temporal bone then going upward and backward involving right parietal and occipital bone.
4	Fissured fracture of length 14 cm involving right middle cranial fossa extending backward to the posterior cranial fossa.
5	Subdural hematoma with a large area of size 15 cm x 10 cm was seen over the right temporal, parietal, and right lateral occipital parts of the brain (Figure 1).
6	Subarachnoid hemorrhage was seen over both hemispheres of the brain area involving 14 cm x 8 cm.
7	Laceration of size 8 cm x 6 cm x 1 cm on the right temporal lobe of the brain.

**Table 2:** Chest injuries

S. No.	Details of injuries
1	Contusion of size 6 cm x 5 cm over the right lateral part of chest situated 15 cm below the mid axillary point.
2	Multiple therapeutic small puncture marks were seen over the anterolateral part of the chest, situated just below and lateral end of the clavicle, an area involving 4 cm x 3 cm, puncture size of 1 cm x 1 cm to 1 cm x 1.5 cm; ecchymosis was seen around the puncture site (Figure 2A).
3	This multiple puncture mark went up to the left atrium; the course of the puncture mark was through skin intercostal muscles, piercing the right pleura and lung (Figure 2B), then the pericardium and the right atrium up to the left atrium, along with the atrioventricular nodal septal area of the heart (Figures 2C, 2D).
4	Contusion was observed in the track of the puncture mark, and the puncture was also appreciated at the lower and posterior sides of the interatrial septal.
5	Abrasion of size 0.5 cm x 0.3 cm over the right anterior part of the shoulder joint situated 5 cm below the tip of the right shoulder joint, which was reddish-brown.
6	Rib numbers 3rd and 4th on the right side were found fractured and separated on the mid-axillary line around the fracture site, and muscle was found contused.
7	The right-side pleura was lacerated under injury no. 06, the right lateral part of the lung middle lobe was found contused and the right chest cavity contained approximately 100 mL of free blood.

On external examination, the following findings were noted: eyes were closed, the cornea was hazy, the mouth was open, the denture was visible, rigor mortis was present all over the body, and postmortem staining was present on the back side of the body and fixed, except contact pressure area. Diffuse swelling was visible over the right side of the scalp and face and a bandage was seen over the right ear. The bandage was also present over the right collarbone area and multiple therapeutic puncture marks were visible beneath it. The body was kept in a dead body cooler of the mortuary overnight.

**Figure 3:**
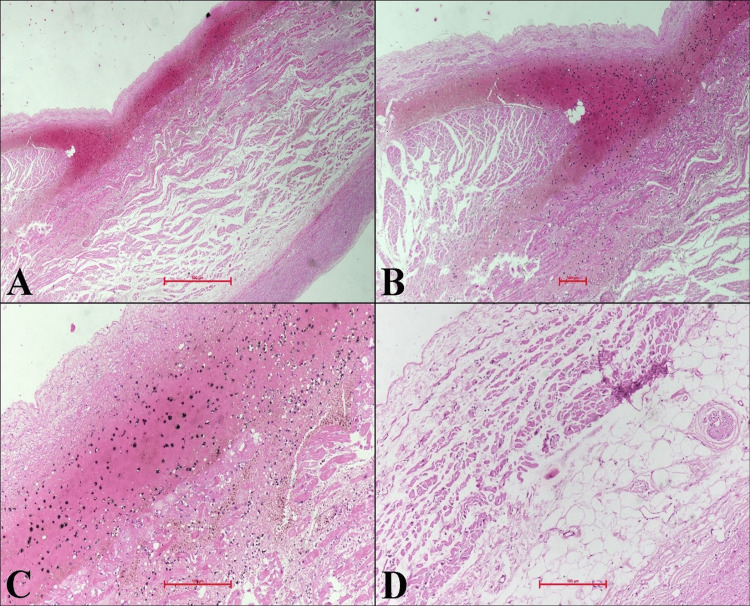
Microscopic pictures (A-B) Microphotographs of the heart showing localized subendocardial hemorrhage myofibril lysis (hematoxylin & eosin (H&E) x40 and x100). Tissue was taken from the atrioventricular area of the heart. (C) Subendocardial area showing fresh and old hemorrhage in the form of blood and hemosiderin-laden macrophages (H&E x400). (D) Subendocardial area showing interstitial edema as marked (H&E x400). Tissue was taken from the interatrial septum from the left atrial side.

Other injuries included abrasion of size 1.5 cm x 0.5 cm over the face, situated 3 cm lateral to and 2 cm below the outer canthus of the right eye and reddish-brown in color. Subendocardial hemorrhages with subendocardial edema were seen in the AV region of the heart during histopathological examination (Figure 3).

The above-mentioned injuries were seen in the cardiac tissue and the microscopic examination report was suggestive of puncture injury to the AV area of the heart.

## Discussion

Various cardiovascular etiologies such as injury to the conductive system and narrowing or constriction of the AV nodal artery steer to sudden death. An examination of the conductive system of the heart can yield diagnostic information about sudden death. An injury to the conductive system of the heart can lead to fatal arrhythmia and death. Arrhythmias cannot be diagnosed through autopsy examination. The cardiac conductive system core is situated just behind the sternum. Contusion of the cardiac conductive system by blunt force trauma also may be a cause of death [5]. Minor injury over the AV node of the conductive system may lead to fatal arrhythmia and can cause death. Subendocardial hemorrhage at the AV nodal area may be considered as the cause of death [7]. Cardiac injuries depend upon the magnitude of force, location of the heart, and nature of the object, which determines the type and pattern of injury resulting in arrhythmias and death [5].

The craniocerebral injuries seen are sufficient to cause death in the ordinary course of nature, but before death, the interventions that were done caused conductive system injuries, and both side walls of the atrial chamber of the heart were also injured. It is noted that multiple injuries to the different heart chambers are directly correlated with mortality [8]. Arrhythmia due to cardiac conductive system injury, which induces sudden death, is not feasible to diagnose. Commotio cordis involves a blunt force blow to the heart area leading to sudden death [9]. Injury to the AV nodal area correlated and confirmed by histopathological examination expressed as petechial hemorrhages over the interatrial septum [5]. At the terminal stage, resuscitation was done, and an observation of the conductive system (nodal area) showed injuries, which were confirmed by histopathological examination. Conductive system injuries sustained during resuscitation are due to fatal techniques.

## Conclusions

Injury to the AV node causes conductive system failure, and finally fatal arrhythmia, which leads to death. Such injuries may also cause sudden death. In the terminal stage, when severe body injuries are sufficient to cause death and conductive system injuries are induced during resuscitation, it may be difficult to determine the cause of death. The examination of the heart during autopsy yielded information about sudden death and it may be in consideration. We conclude that conductive system injury causes death induced by fatal arrhythmia.
